# The Many Faces of Intelligence: A Discussion of Geary’s Mitochondrial Functioning Theory on General Intelligence

**DOI:** 10.3390/jintelligence8010008

**Published:** 2020-02-17

**Authors:** Paul De Boeck, Kristof Kovacs

**Affiliations:** 1 Department of Psychology, Ohio State University, Columbus, OH 43210, USA; 2 ELTE Eötvös Loránd University, 23-27, Kazinczy Street, 1075 Budapest, Hungary; kovacs.kristof@ppk.elte.hu

David Geary’s article on intelligence (Geary 2018) and the summary of his theory in *Journal of Intelligence* offer a refreshing and inspirational view on intelligence. One of the longest standing issues in intelligence research is across-domain variance: why do tests with seemingly different contents covary? Is there a casual mechanism common to all these tests? Geary lays out a multi-layer theory of general intelligence, starting with mitochondria-based cellular energy at the deepest level, a factor that affects all cognitive activity. Yet, in his theory there is also room for various mechanisms at higher levels, including brain networks, up to cognitive processes underlying intelligence test performance. Moreover, the theory explains links of general intelligence with other variables such as health. It is a fresh way of thinking about general intelligence.

Per layer in Geary’s concentric representation of intelligence there is a different face of intelligence and, in fact, many different faces closer to the outer layer. Geary’s view combines a diversity of aspects which are often considered as contradictory and inconsistent, whereas, in fact, they are not. There is a single underlying kind of mechanism (cellular energy) but higher up, towards the behavioral level, there also is a large diversity of mechanisms to produce the intelligence that can be observed in problem solving performances. There is plenty of room for learning and a diversity of individual differences depending on how cellular energy translates into behavior through intermediate layers, while, at the same time, an explanation of g is also offered. In the following, we suggest some possible topics for discussion. 

A first point concerns the definition of intelligence. Perhaps we do not need a definition of intelligence to investigate intelligence. For example, it is worth investigating interlayer relationships and mechanisms for a broad domain of tasks. Different theories at different levels can be true at the same time. It is of interest that a common underlying source does not imply a unitary concept of intelligence at the level of problem-solving even though the underlying principle may explain the positive manifold. 

A second point of possible interest for discussion is the opposition between reflective and formative models. While it is a nice and elegant model opposition, the reality is perhaps more complex, based on the following interpretation of Geary’s theory. The production of cellular energy as a ceiling concept and the way it trickles down (trickles up?) to higher layers is inconsistent with the simple linear relationships implied by reflective and formative models, which commonly focus on the outer layer of problem solving. Below the ceiling, there is room for how the energy is used through the layers which lead to performance, and beyond the ceiling there is room for strategical mechanisms to circumvent energy limitations. Formative conceptualizations can still be used to roughly summarize a variety of upper layer performance of various kinds and as underpinnings of approximate domain measures of problem solving. However, the mechanisms described in Geary’s article may be too complex for the existing psychometric models, which work with fixed and linear relationships between variables. In practice, they can still be summative approximations with predictive value for other variables. 

Third, Geary’s theory also seems to have interesting links with very early theories of intelligence. The mitochondrial energy notion of intelligence can perhaps be interpreted as nicely fitting with the theories of the earliest empirical psychologists such as James McKeen Cattell, Wundt, and Spearman, and their concept of intelligence as mental energy. At the same time, it also fits with Wechsler’s (1975) view that intelligence cannot be defined and that “Intelligence has no invariants, but its components sometimes act as if it does” (p. 138), as well as with Eysenck’s (1979) distinction of biological and psychometric intelligence. One would need to go beyond intelligence performance, from the upper layers to the bottom layer, to find an invariant in the form of mitochondrial functions. Although unitary in kind, the cellular energy is differentially distributed across cells, with somewhat different levels across the brain and different ways in which the energy may play a role. 

In sum, Geary’s theory proposes a unitary kind of biological mechanism underlying general intelligence. Based on possible interpretations of his theory, several questions can be raised regarding existing conceptualizations of intelligence. Some of these questions are formulated in this editorial. We are sure there are more such questions. We are looking forward to an inspiring discussion.
